# Hydrogen Sensing Using Paper Sensors with Pencil Marks Decorated with Palladium

**DOI:** 10.3390/s19143050

**Published:** 2019-07-10

**Authors:** Nam Hee Lee, Un-Bong Baek, Seung-Hoon Nahm

**Affiliations:** 1 Department of Chemistry, KAIST, 291 Daehak-ro, Yuseong-gu, Daejeon 34141, Korea; 2 Energy Materials Metrology, Korea Research Institute of Standards and Science, 267 Gajeong-ro, Yuseong-gu, Daejeon 34113, Korea

**Keywords:** hydrogen, H_2_ sensing, paper-based sensor, pencil marks, palladium, chemiresistor

## Abstract

Paper-based sensors fabricated using the pencil-on-paper method are expected to find wide usage in many fields owing to their low cost and high reproducibility. Here, hydrogen (H_2_) detection was realized by applying palladium (Pd) nanoparticles (NPs) to electronic circuits printed on paper using a metal mask and a pencil. We confirmed that multilayered graphene was produced by the pencil, and then characterized Pd NPs were added to the pencil marks. To evaluate the gas-sensing ability of the sensor, its sensitivities and reaction rates in the presence and absence of H_2_ were measured. In addition, sensing tests performed over a wide range of H_2_ concentrations confirmed that the sensor had a detection limit as low as 1 ppm. Furthermore, the sensor reacted within approximately 50 s at all H_2_ concentrations tested. The recovery time of the sensor was 32 s at 1 ppm and 78 s at 1000 ppm. Sensing tests were also performed using Pd NPs of different sizes to elucidate the relationship between the sensing rate and catalyst size. The experimental results confirmed the possibility of fabricating paper-based gas sensors with a superior sensing capability and response rate.

## 1. Introduction

Hydrogen (H_2_) has immense potential as an environmentally friendly resource, and can provide potential solutions to issues such as fossil fuel depletion and environmental pollution. Hydrogen, owing to its high energy content and clean nature, is considered an ideal future fuel and is highly promising as an energy source in many industrial and technical applications. However, hydrogen concentrations of more than 4 vol% in air are potentially explosive [1,2]. For instance, hydrogen present in concentrations of 15%–59% can cause an explosion at atmospheric pressure [1,2,3,4]. Moreover, as hydrogen gas is colorless and odorless, the development of high-performance H_2_ monitoring systems and leak detection sensors has become essential.

Currently studied hydrogen sensors include those based on catalytic combustion or a hot wire, oxide films, and bipolar-structured Schottky barrier diodes. However, these have limitations related to their large sizes, complicated structures, and high costs, as well as their high operating temperatures and power consumption [5,6,7,8,9,10]. In addition, while hydrogen sensors based on metal oxides such as SnO_2_, In_2_O_3_, ZnO, NiO, and TiO_2_ show high sensitivities, they operate at approximately 400 ℃, which is disadvantageous in terms of power consumption [11,12,13,14,15,16,17].

Palladium is an attractive hydrogen-sensing material that shows high hydrogen selectivity at room temperature owing to its excellent ability to adsorb hydrogen at room temperature. However, it has been reported that hydrogen gas sensors based on Pd films may be vulnerable to structural changes that result from the formation of palladium hydride (PdHx) at hydrogen concentrations greater than 2% [18,19,20,21].

Phase transitions in Pd films with changes in hydrogen concentration can cause irreversible swelling and mechanical damage, and affect their hydrogen diffusion coefficients [22,23]. In particular, a low hydrogen diffusion coefficient (3.8 × 10^−7^ cm^2^/s at 298 K) can lead to long response times at low hydrogen concentrations [23,24]. This problem can be solved by applying Pd particles to other nanostructures in order to increase the surface area [25,26,27] or by developing Pd nanostructures such as nanowires [28,29,30,31], nanotubes [32], and nanocomposites [33].

Owing to their attractive characteristics, such as their excellent sensitivities, stabilities, and low power consumptions, Pd nanostructures are being studied extensively for use in hydrogen sensors. In addition, graphene is an attractive material for many applications because it has a larger surface area compared to the other above-mentioned dimensional structures.

Johnson et al. reported hydrogen sensing with a sensitivity of 55% at a minimum concentration of 40 ppm and were able to achieve a 50% response time of 21 s and a 50% recovery time of 23 s from a multilayered-graphene-based nanoribbon network functionalized with Pd using e-beam evaporation [34]. Martinez-Orozco et al. realized hydrogen detection based on Pd nanoparticles (NPs) immobilized on graphene oxide (GO) synthesized by the microwave ablation method [35]. They reported a 90% response time of less than 1 min for hydrogen concentrations of 0.01–5 vol% in the atmosphere and a 90% recovery time of less than 5 min at low concentrations.

Duy-Thach et al. developed sensors using Ni [36] and Pd nanocubes [37] for Pd-reduced graphene oxide (RGO). These sensors exhibited distinct shark-fin-like signals, indicating slow adsorption and desorption kinetics with respect to H_2_. Kim and Jung et al. fabricated a gas sensor by depositing Pd on the surfaces of graphene nanoribbons such that the sensor was devoid of polymer residues that can negatively affect the sensitivity of the sensor. This graphene-nanoribbon-based sensor could detect hydrogen at concentrations of 30–1000 ppm and exhibited a 90% response time of less than 60 s at 1000 ppm and an 80% recovery time of less than 90 s [38]. Previously reported carbon materials-based sensors for hydrogen detection are summarized in Table 1. 

The graphene-based hydrogen gas sensors mentioned above require the careful handling of graphene as well as complex fabrication processes. Furthermore, in the case of GO- and RGO-based sensors, the recovery signal can be slow and irreversible because defects in the graphene layers and the oxygen functional groups present on the graphene surface act as active sites for interactions with gas molecules [39].

However, as two-dimensional carbon materials are still attractive as supports for metal NPs [39], it is necessary to develop a simple and easily fabricable carbon-based sensors.

In previous reports on the production of paper-type sensors made by pencil drawing, Zhang et al. fabricated variable resistors and strain sensors using the pen-on-paper method [40], while Zhang and Ma et al. developed a temperature-responsive paper-based sensor using pencil lead containing an ionic liquid [41]. Furthermore, Huang et al. fabricated NO_2_ gas sensors based on pencil drawings and Ag electrodes made on paper [42]. These sensors showed a sensitivity of 8.34% and fast response and recovery signals (response time of 71 s and recovery time of 25 s) at 5 ppm NO_2_.

In this paper, we describe the fabrication of a paper-based H_2_ sensor that is inexpensive to produce and shows superior sensitivity at room temperature; we also experimentally evaluated the performance of the sensor. Pd NPs [43] were used to fabricate sensors with low noise and high sensitivity.

The hydrogen-sensing ability of the sensor is based on the change in its resistance in response to exposure to and shielding from hydrogen. Evaluation testing confirmed the excellent performance of the sensor, in terms of its high sensitivity and low response time.

## 2. Materials and Methods

### 2.1. Sensor Fabrication

**Figure 1:**
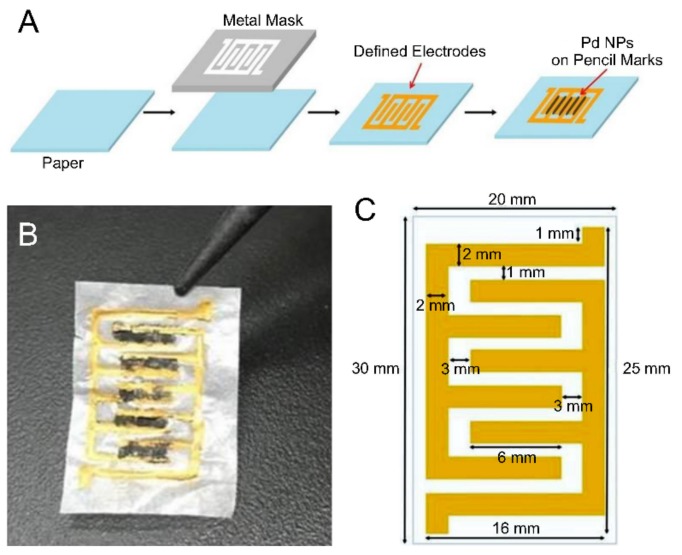
(**A**) Process for fabricating a paper-based sensor for hydrogen detection. (**B**) Photographic image of the fabricated sensor. (**C**) Schematic of the paper-based sensor. The gas sensor was produced by a metal-stencil printing technique and drawing with a 4B pencil.

The fabrication of the gas sensor started with the printing of the electrodes on paper. Electrodes were formed on 30 mm × 20 mm weighing paper (HS120116, Heathrow Scientific, USA) using the metal-stencil printing technique, which is mainly used with surface-mounted technology. The structure of each electrode, which was printed using Au paste, resembled entwined fingers. The Au electrodes were 16 mm long, 2 mm wide, and 20 μm thick (Figure 1c).

Next, a chemiresistor-type hydrogen sensor was fabricated on paper using a 4B pencil and Pd NPs. Au electronic leads, separated from each other to allow for a perfect circuit configuration, were connected by the mechanical wear of the pencil. The most common type of pencil (4B) was used. The dimensions of a single active area were 6 mm × 1 mm, and the sensor had five such active areas. The average resistance of fabricated sensors was about 63 kΩ.

Next, Pd NPs were added onto the graphene layers exfoliated from the pencil to increase their selectivity with respect to hydrogen gas. The Pd NPs (PlasmaChem, Germany), which had an average size of 6–7 nm, acted as the catalyst for hydrogen sensing. 

Finally, 2 μL of a 1 wt% dispersion of the Pd NPs in hexane, produced by centrifugation (500 rpm, 3 min, 3 times), was added in a dropwise manner to the active area. Pencil marks were then formed on top of the metal NPs for coverage. This process was repeated several times such that a total of 10 μL of the Pd NP solution was added to each active area.

Other Pd NPs used as catalysts had average sizes of 22 nm (Sigma-Aldrich, St. Louis, MO, USA) and 52 nm (American Elements, Los Angeles, CA, USA).

### 2.2. Sensor Testing

Hydrogen detection tests were performed using a laboratory-based sensor testing system. The sensing system consisted of a 15 L cylindrical stainless steel chamber, a probe station, a gas flow controller, a voltage source (7651 programmable DC source, Yokogawa, Musashino, Tokyo, Japan), and a multimeter (34401A digital multimeter, Hewlett Packard, Palo Alto, CA, USA). 

The fabricated sensor was placed in the probe station of the stainless steel chamber, and a fixed voltage of 1 V was then applied to the sensor. The electrical signal generated in response to exposure to H_2_ gas was observed using LabView software. The hydrogen exposure experiments started with the injection of high-purity (99.999%) N_2_ into the chamber for 15 min at a flow rate of 2000 sccm to establish a stable electrical signal and to exclude other reactive gas molecules. Once the sensor exhibited a stable response, H_2_ was injected into the N_2_ flow, and the resulting signal of interest was detected. After a signal was recorded, the gas-detection chamber was purged with N_2_ to allow the sensor to recover.

All experiments were carried out at room temperature under the following conditions: relative humidity, 30%; pressure, 1 bar; and total gas flow rate, 5000 sccm. The flows of all gases were controlled with a flow controller.

### 2.3. Characterization of Graphene Sheets

Raman spectroscopy (LabRAM HR, Horiba, Minami, Kyoto, Japan) was performed using a 532-nm Ar-ion laser to characterize the graphene sheets produced by the mechanical abrasion of the pencil. The presence of the Pd NPs used for the detection of the gas molecules was confirmed by X-ray photoelectron spectroscopy (XPS, Sigma Probe, Thermo VG Scientific, Waltham, MA, USA) and X-ray diffraction (XRD, SmartLab, RIGAKU, Akishima, Tokyo, Japan). The XRD patterns were acquired over the Bragg angle range of 5–80° at a rate of 2°/min and a step size of 0.01°.

Field-emission scanning electron microscopy (FESEM, Quanta FEG 650, Thermo Scientific Waltham, MA, USA,) and field-emission transmission electron microscopy (FETEM Talos F200X, FEI, Hillsboro, OR, USA) were used to analyze the sensor surface.

Furthermore, the graphitic layer and Pd NPs used to form the sensor were also subjected to thermogravimetric analysis (TGA, TG209 F1 Libra, Netzsch, Germany), which was performed at temperatures ranging from room temperature to 800 °C. The temperature was raised in a stepwise manner at a rate of 10 °C/min, and these experiments were performed under a flow of air.

## 3. Results and Discussion

The fabrication process, a schematic, and a photographic image of the paper-based gas sensor are shown in Figure 1. Nonconductive paper becomes conductive due to the printed Au and the pencil marks. In particular, detection of the target occurs at the surfaces of the pencil marks.

**Figure 2:**
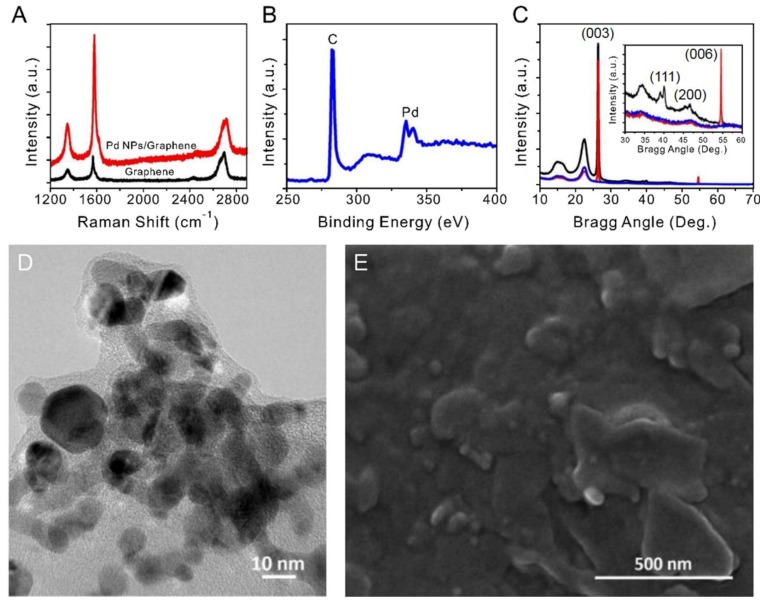
Characterization of the multilayered graphene sheet produced by pencil and confirmation of the presence of Pd NPs (**A**) Raman spectra of a multilayered graphene sheet and a multilayered graphene sheet decorated with Pd NPs. (**B**) XPS profile of the active area of a sensor. (**C**) XRD patterns of the active area of a sensor: plain paper (blue), multilayered graphene sheet/paper (red), and Pd NPs/multilayered graphene sheet/paper (black) were compared to confirm the presence of Pd NPs. Inset: enlarged spectra in the Bragg angle range of 30–60° used to identify Pd. (**D**) TEM image used to determine the size of Pd NPs. (**E**) SEM image showing morphologies of the graphene layer and Pd NPs; 6–7-nm Pd NPs are scattered widely between the multilayered graphene sheets.

The Raman spectrum of the pencil marks made directly on paper is shown in Figure 2a. The spectrum consists of a D band at 1346 cm^−1^, a G band at approximately 1564 cm^−1^, and a 2D band at approximately 2695 cm^−1^. The intensity of the G band is higher than that of the D band. These results are consistent with those of Wang et al. who reported that the intensity of the D band of natural graphite is much lower than that of the G band, and that the intensity of the D band decreases as the number of graphene layers is increased [44]. The I_2D_/I_G_ intensity ratio is 1.15, while the full width at half maximum of the 2D band is 64 cm^−1^.

The Raman spectra confirm that multilayered graphene sheets were formed on the paper by the mechanical abrasion of the pencil [45]. In addition, higher-intensity D and G peaks were observed in the Raman spectrum of the graphene sheet containing the Pd NPs compared to the spectrum of the graphene sheet alone.

The presence of Pd NPs on the graphitic sheet was confirmed by XPS and XRD (Figure 2b,c). The XPS profile of the graphene sheet coated with Pd NPs exhibited double peaks at 335.01 eV and 340.87 eV typical of Pd [46]. The XRD pattern consisted of peaks at 26° and 55°, which correspond to graphene, as well as peaks at 40.1° and 46.6°, which correspond to Pd with a face-centered cubic structure. Owing to the large amount of multilayered graphene in the sensor, the relative intensities of the peaks of the Pd NPs loaded on the surface of the active region were low.

Figure 2d,e shows TEM and SEM images of the Pd NPs located between the graphene layers. The Pd NPs were determined to have an average size of 6–7 nm. Finally, the SEM image confirmed that the Pd NPs were well-dispersed between the graphene layers and did not undergo significant aggregation.

In order for a sensor functionalized with Pd NPs to exhibit fast gas adsorption/desorption dynamics and low ohmic resistance, it is essential that the Pd NPs are well-dispersed within the electrically conductive path. In addition, surface analysis revealed that layered structures of graphite flakes originating from the pencil were clearly formed directly on the paper [44,47,48].

**Figure 3:**
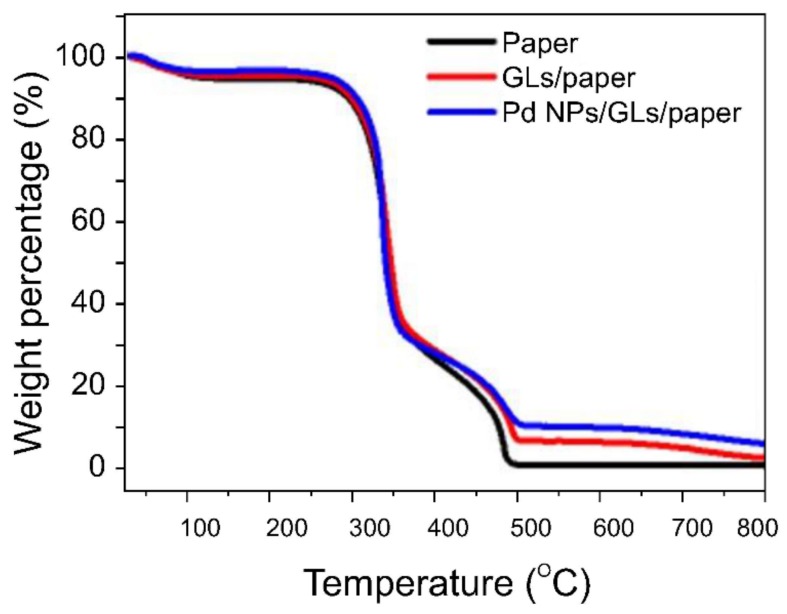
TGA traces used to quantify the number of NPs present in the sensor. The number of Pd NPs located on graphitic layers (GLs) was determined from the residue at approximately 800 °C, at which point sample pyrolysis was complete.

TGA is a useful method for determining the amounts of metallic materials contained in carbon-based materials, and was used in this study to determine the number of Pd NPs actually participating in hydrogen detection (Figure 3).

TGA was performed on plain weighing paper, multilayered graphitic sheet/weighing paper, and Pd NPs/multilayered graphitic sheet/weighing paper at temperatures ranging from room temperature to 800 °C. Because weighing paper was used as the substrate, the decomposition processes for the three samples were similar.

The Pd-NP content in the final residue was approximately 3.41% at 799 °C; that is to say, approximately 60 µg of Pd NPs was present in the multilayered graphene. 

The detection of hydrogen is realized by variations in the resistance of the sensor after it comes in contact with hydrogen molecules, and the electrical response of the sensor is related to its sensitivity. The sensitivity (*S*) of a sensor is defined as:

(1) $$S\left(\%\right)=\frac{\left(R_{H}-R_{i}\right)}{R_{i}}\times 100$$

where *R_H_* is the resistance of the sensor after exposure to hydrogen gas and *R_i_* is the initial resistance of the sensor. The recovery rate of the sensor was measured by starting and stopping the supply of H_2_. The recovery of a hydrogen gas sensor in a N_2_ atmosphere is slow and unstable. Hence, no recovery-promoting gas was used so that the natural removal of hydrogen gas from the fabricated sensor could be evaluated.

**Figure 4:**
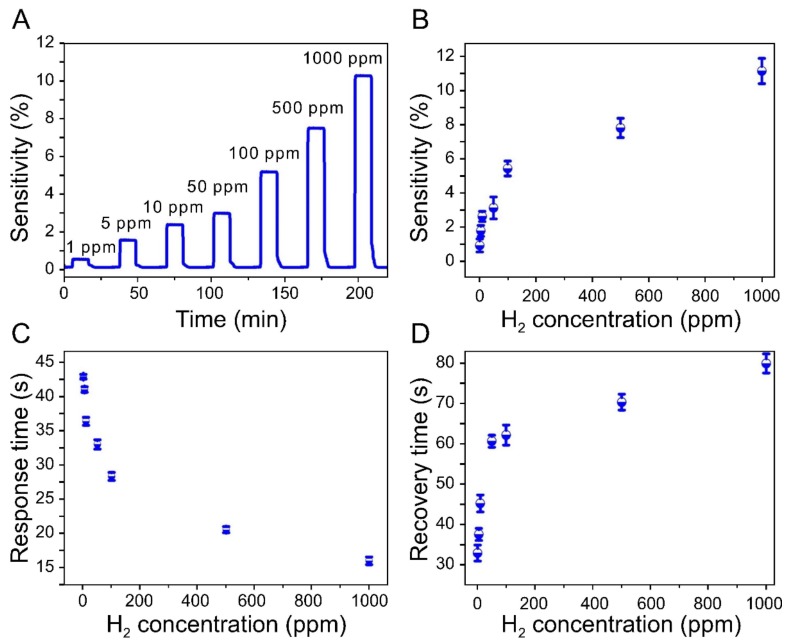
Dynamic response of the sensor to changes in hydrogen concentration. (**A**) Sensitivity results at 1, 5, 10, 50, 100, 500, and 1000 ppm H_2_. (**B**) Comparison of sensitivities at seven different concentrations. (**C**) Sensor response times at various concentrations of hydrogen. (**D**) Sensor recovery time as a function of hydrogen concentration. The experimental data indicate that the sensor has excellent hydrogen-detection capabilities (detection limit of 1 ppm) with fast response times.

The dynamic response of the sensor at seven different hydrogen concentrations (1, 5, 10, 50, 100, 500, and 1000 ppm) is shown in Figure 4. These measurements were performed by interrupting the hydrogen supply after the sensitivity had plateaued for approximately 10 min.

Once the detection signal had decreased to that corresponding to the initial resistance due to hydrogen desorption, the sensitivity of the sensor at another concentration was immediately measured.

Here, the response time refers to the time taken by the sensor, from the appearance of the detection signal, to reach 90% of the saturation value, while the recovery time implies the time during which 90% of the changed signal is recovered from the interruption of the gas supply. The electrical signal in response to the target gas was observed immediately irrespective of the gas concentration.

When hydrogen molecules come in contact with the Pd NPs, dissociative adsorption of hydrogen occurs on the surfaces of the NPs, resulting in a decrease in the work function of Pd. Consequently, electrons are transferred to the graphene sheet, which acts as a conductive path between the source and the drain of the sensor, thereby increasing the resistance because the primary carriers of graphene, namely holes, are depleted [49,50,51]. Therefore, the detection mechanism is attributable to the reversible adsorption of H_2_ on the Pd NPs. When N_2_ is used instead of H_2_, the partial pressure of H_2_ decreases, and all the hydrogen atoms are desorbed, resulting in a decrease in resistance.

Figure 4a shows the electrical behavior of the sensor in response to the adsorption and desorption of hydrogen at different concentrations and confirms the high sensitivity of the sensor, which detected H_2_ even in very low concentrations (as low as 1 ppm).

The maximum sensitivity of the sensor with respect to each concentration of hydrogen remained stable during these measurements, and the resistance of the sensor decreased to its initial value in every case after the natural removal of the adsorbed hydrogen.

The rates of adsorption (*r_a_*) and desorption (*r_d_*) of gas molecules are defined as follows [52]:

*r_a_* = *K_a_C*(1 − *θ*)^2^, *r_d_* = *K_d_θ*^2^ (2)

Here, *K_a_* and *K_d_* represent the adsorption and desorption rate constants, *C* is the hydrogen concentration, and *θ* is the surface fraction available for hydrogen adsorption. The initial response of the sensor is linearly related to the hydrogen concentration as the fraction of the surface occupied by hydrogen molecules is initially negligibly small.

However, the recovery rate is substantially lowered at relatively higher *θ*^2^ values, resulting in a detection curve that resembles a shark fin [27,38,53]. On the other hand, the fabricated sensor exhibited square response curves, rather than shark-fin-like curves, even at very low hydrogen concentrations, which is probably attributable to the large surface-area-to-volume ratio of the Pd NPs, which were less than 10 nm in size and therefore evenly dispersed between graphene layers.

Figure 4b shows the relationship between the sensitivity of the sensor and the hydrogen concentration. As can be seen from the figure, the relationship is not completely linear. However, the sensitivity increases with an increasing hydrogen concentration. This relationship is consistent with Sieverts’ law, which is applicable for low concentrations of hydrogen [18,54]:

(3) $$S\propto \frac{H}{Pd}=\frac{1}{K_{s}}{\left(pH_{2}\right)}^{\frac{1}{2}}$$

where *H*/*Pd*, which is defined as the ratio of the number of hydrogen atoms to the number of Pd atoms in the Pd-H system, is proportional to sensitivity, while *K_s_* is the Sieverts constant and *pH*_2_ is the hydrogen partial pressure. This rule is valid for H_2_ concentrations of up to 10,000 ppm in the Pd-H system [23,35]. Therefore, we conclude that the experimental results follow Sieverts’ law and that the characteristics of hydrogen adsorption on the surfaces of the Pd NPs can be explained by that law. 

The experimental relationships between the reaction rates of the sensor and the gas concentration are shown in Figure 4c,d. The response time to reach 90% of the saturation signal decreased with increasing hydrogen concentration. Nevertheless, the sensor responded within approximately 50 s at all concentrations. The time required for the resistance of the sensor to decrease to the initial level, which is driven by the desorption of H_2_, increased with increasing gas concentration.

The recovery time was 32 s at the lowest concentration of 1 ppm, while at the highest concentration of 1000 ppm, it took 78 s to recover 90% of the total change in sensor resistance. Even at the highest concentration, the recovery time was within 2 min. The obtained experimental results highlight the superior reaction rate of the paper-based sensor with respect to the target gas and confirm that the Pd NPs were uniformly dispersed within the graphene layers. 

**Figure 5:**
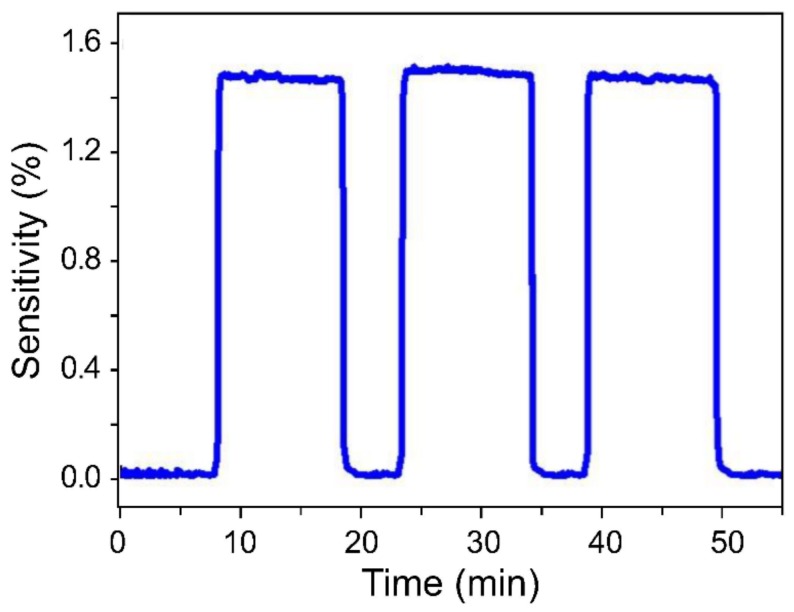
Sensor-response repeatability at constant H_2_ concentration. The cycle included a response time of 10 min and a recovery time of 5 min and was repeated five times at 5 ppm.

In order to evaluate sensor reproducibility and stability, the sensor was subjected to three measurement cycles, with each cycle consisting of 10 min on-state and 5 min off-state periods at 5 ppm (Figure 5). Even though these measurements were performed at a low concentration, the sensor showed quick and stable on/off switching characteristics as well as constant sensitivity during each cycle. Furthermore, the sensor exhibited a response time of 41 s and a recovery time of 36 s, and its response curve was not shark-fin shaped, as was the case during slow recovery.

The sizes of the Pd particles used in the gas-sensing catalyst determine the reaction rate of the sensor. As per Sieverts’ law, the two factors that determine the value of K_s_ are the effective exposure area of the Pd layer and the depth of hydrogen-atom penetration into the Pd layer [22].

**Figure 6:**
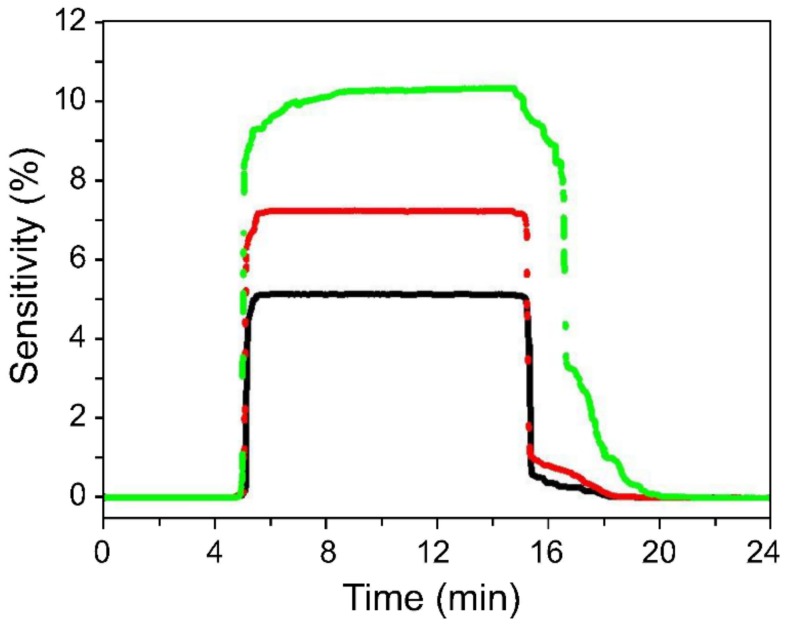
Sensor sensitivity of as function Pd NP size. Sensor response and recovery rates improved with decreasing NP size. The black, red, and green response curves were produced with <10 nm, <25 nm, and <60 nm Pd NPs, respectively.

Real-time H_2_ detection was performed at a concentration of 100 ppm using sensors composed of 6-nm, 22-nm, and 52-nm Pd NPs (Figure 6). The on-state condition was maintained for approximately 10 min after the commencement of gas injection, and the natural removal of the hydrogen molecules was allowed to occur immediately.

Figure 6 reveals that the response and recovery times of the sensor increased with increasing Pd NP size. The time taken for the hydrogen atoms to be removed from the largest-diameter Pd NPs exceeded 3 min, whereas the recovery time for NPs smaller than 10 nm was 60 s.

A sensor with large catalyst particles will exhibit a higher sensitivity as it contains a greater number of free sites onto which hydrogen atoms can be adsorbed; however, the sensor will take longer to reach saturation. In addition, a longer recovery time than response time is required for the hydrogen atoms deeply diffused into the Pd to completely escape from the surface of the Pd particles. In agreement with these expectations, the experimental results revealed that hydrogen atoms diffused deep into the Pd require longer times to completely escape from the Pd surface when larger particles are used.

## 4. Conclusions

In conclusion, we demonstrated a simple and inexpensive method for fabricating paper-based sensors that detect H_2_ at room temperature. The hydrogen sensors were constructed by printing electrodes on a piece of paper, marking the paper with a 4B pencil, and then coating the pencil marks with 10-nm or smaller Pd NPs. The fabricated sensors exhibited reliable and repeatable sensing characteristics, reacting quickly to both exposure to hydrogen and its removal. These sensors, which have excellent sensing abilities and high response rates, highlight the potential of pencil-and-paper-based devices.

## Figures and Tables

**Table 1:** Carbon materials-based sensors for H_2_ sensing.

Materials	H_2_ Concentration (ppm)	Work Temperature	Sensitivity (%)	Response Time *	Recovery Time **
SWCNT [27]	100 (in air)	RT	ΔR/R_0_ = 0.4	18 min (90%)	20 min (90%)
GNR network [34]	40 (in air)	RT	($R_{H_{2}}$ − *R*)/*R* = 55	21 s (50%)	23 s (50%)
GO [35]	5000 (in air)	RT	(*R_g_* − *R_a_*)/*R_a_* = 13	30 s (90%)	7 min (90%)
GNR array [38]	1000 (in N_2_)	RT	(*R_s_* − *R_i_*)/*R_i_* = 3	60 s (90%)	90 s (80%)
GLs (Our work)	1 (in N_2_)	RT	(*R_H_* − *R_i_*)/*R_i_* = 1	42 s (90%)	32 s (90%)

Abbreviations in Table 1: SWCNT (single-wall carbon nanotube); GNR (graphene nanoribbon); GO (graphene oxide); GLs (graphitic layers); RT (room temperature). * Response time: The time to reach a certain level of saturation value. ** Recovery time: The time required for a certain level of the changed electrical signal to recover.
